# New and Emerging Targeted Therapies for Advanced Breast Cancer

**DOI:** 10.3390/ijms23042288

**Published:** 2022-02-18

**Authors:** Kristie H. Lau, Alexandra M. Tan, Yihui Shi

**Affiliations:** Department of Basic Science, College of Medicine, California Northstate University, Elk Grove, CA 95757, USA; kristie.lau8761@cnsu.edu (K.H.L.); alexandra.tan8903@cnsu.edu (A.M.T.)

**Keywords:** breast cancer, breast cancer treatment, HER2, targeted therapy, emerging therapies

## Abstract

In the United States, breast cancer is among the most frequently diagnosed cancers in women. Breast cancer is classified into four major subtypes: human epidermal growth factor receptor 2 (HER2), Luminal-A, Luminal-B, and Basal-like or triple-negative, based on histopathological criteria including the expression of hormone receptors (estrogen receptor and/or progesterone receptor) and/or HER2. Primary breast cancer treatments can include surgery, radiation therapy, systemic chemotherapy, endocrine therapy, and/or targeted therapy. Endocrine therapy has been shown to be effective in hormone receptor-positive breast cancers and is a common choice for adjuvant therapy. However, due to the aggressive nature of triple-negative breast cancer, targeted therapy is becoming a noteworthy area of research in the search for non-endocrine-targets in breast cancer. In addition to HER2-targeted therapy, other emerging therapies include immunotherapy and targeted therapy against critical checkpoints and/or pathways in cell growth. This review summarizes novel targeted breast cancer treatments and explores the possible implications of combination therapy.

## 1. Introduction

Breast cancer (BC) is one of the most commonly diagnosed cancers in women in the United States. One in 8 women are expected to be diagnosed with invasive breast cancer within their lifetime. Breast cancer made up an estimated 30% of all new cancer cases for women in 2021. Over 15% of breast cancer cases in women lead to death, making breast cancer the second most common cause of cancer death in women. The death rate of breast cancer in the United States has been reduced by 41% since 1989; however, the downward trend has recently slowed, emphasizing the importance of new breast cancer treatment discovery [1,2].

BC is a heterogeneous disease that is molecularly categorized by the expression of specific hormone receptors, as well as the overexpression of human epidermal growth factor receptor 2 (HER2) [3]. Breast cancer is often referred to in four major subtypes: HER2, Luminal-A (LumA), Luminal-B (LumB), and Basal-like [4]. LumA, the most frequent BC subtype, is characterized by estrogen receptor (ER) and progesterone receptor (PR) positivity. LumB, like LumA, is ER+ and PR+ but it is differentiated by its high expression of proliferation markers, specifically Ki67. HER2 BC is classified by the overexpression of the tyrosine kinase family HER2 receptor. Finally, basal-like BC is classified as triple-negative (negative for PR, ER, and HER2 receptors). The LumA subtype has the best prognosis of all four subtypes [5].

Further classification of breast cancer includes grade and stage. The Centers for Disease Control and Prevention recommended stages for invasive breast cancer include localized, regional, and distant stages, with 5-year survival rates ranging from 98% to 85% to 30%, respectively, based on stage [6]. Breast cancer grading relies on pathological classification using the Elston–Ellis grading system which accounts for the mitotic count, nuclear pleomorphism, and tubule count [7]. Due to the heterogeneity of breast cancer, it remains imminent that therapies and treatments become increasingly targeted to address the subtype, stage, and grade of breast cancer [8]. Traditional treatments for breast cancer include surgery, chemotherapy, and radiation therapy. Personalized therapy for hormone receptor-positive breast cancer has been well established, including selective estrogen receptor modulators and degraders (SERMs and SERDs), aromatase inhibitors (AI), and endocrine therapy. More recent investigation has revealed improved therapies targeting other subtypes such as triple-negative breast cancer (TNBC) and HER2-positive BC [8].

New emerging technologies are on the horizon for the treatment of breast cancer, including immuno-oncolytic virus drugs, histone deacetylase inhibitors, and novel combination therapies [9,10,11]. In this review, we will discuss the most novel breast cancer treatments for HER2-positive, HER2-negative, and triple-negative breast cancers, and the possibilities implicated in combinatorial therapy for the future of breast cancer medicine (Table 1).

## 2. Targeted Therapies for Endocrine Therapy Resistance

**Figure 1:**
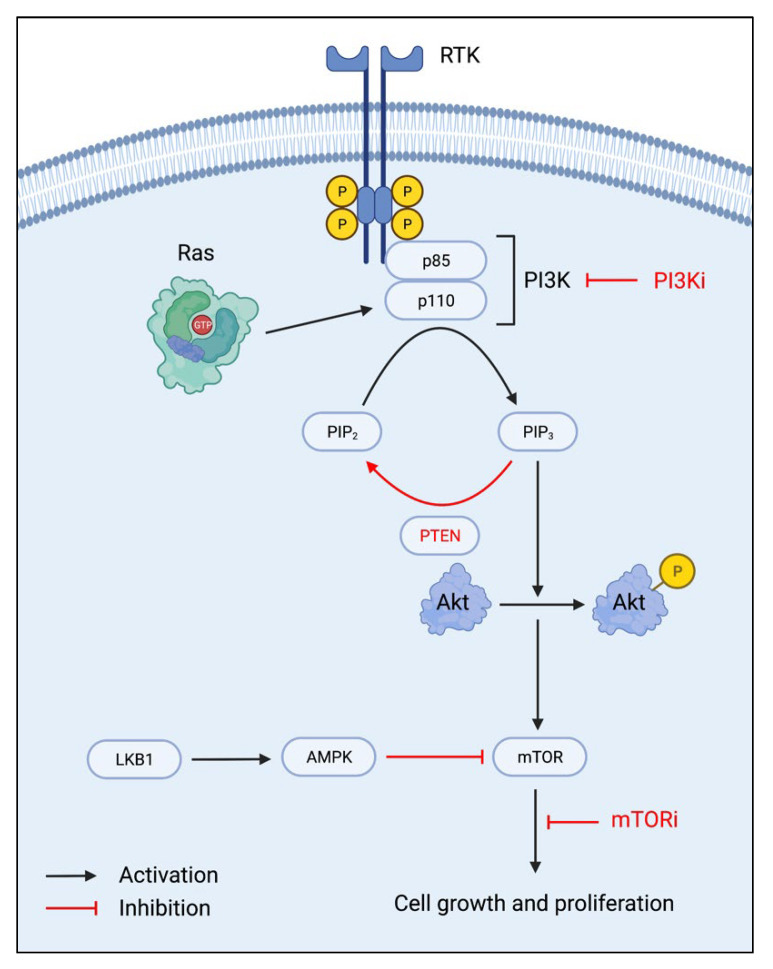
The PI3K/Akt/mTOR pathway and mechanisms of inhibition. RTK: receptor tyrosine kinase; PI3K: phosphoinositide 3-kinase; PI3Ki: PI3K inhibitor; PIP_2_: phosphatidylinositol-4,5-biphosphate; PIP_3_: phosphatidylinositol-3,4,5-triphosphate; PTEN: phosphatase and tensin homolog; Akt: protein kinase B; LKB1: liver kinase B1; AMPK: adenosine monophosphate-activated protein kinase; mTOR: mammalian target of rapamycin; mTORi: mTOR inhibitor.

Three protein components—phosphoinositide 3-kinase (PI3K), protein kinase B (Akt), and the mammalian target of rapamycin (mTOR) complexes, are primary regulators of the PI3K/Akt/mTOR (PAM) pathway, which is associated with cell growth, survival, and proliferation. Due to increased endocrine therapy resistance with increased activation of the PAM pathway, targeting this pathway has been widely researched in cancer treatment for endocrine-resistant breast cancers. PI3K is a heterodimer composed of two subunits, a regulatory p85 subunit, and a catalytic p110 subunit. With receptor tyrosine kinase (RTK) stimulation, the phosphorylated tyrosine residues become a docking site for the p85 subunit. The p110 subunit is subsequently recruited and activated by Ras proteins, thus leading from phosphatidylinositol-4,5-biphosphate (PIP2) phosphorylation to phosphatidylinositol-3,4,5-triphosphate (PIP3) (Figure 1). Akt, a serine/threonine kinase, is then phosphorylated, which leads to the downstream activation of the mTORC1 and mTORC2 complexes. MTOR activation increases cell anabolic growth, primarily through increased protein synthesis for cell growth and proliferation [12,13]. In breast cancer, activating mutations in PI3K and/or aberrant signaling in the absence of growth factors may contribute to its continued proliferation, even in the presence of endocrine therapy.

Thus, therapies targeting inhibiting PI3K, Akt, and mTOR activation have been under research and development to prevent cell cycle progression through the PAM pathway. With mTOR being the prime modulator of cell growth and proliferation, mTOR inhibitors are a special drug category due to their direct effects on the cell cycle. In addition to the inhibition of downstream signaling, inhibitors of PI3K and Akt are also under extensive research since the inhibition of upstream signaling also modulates mTOR regulation of the cell cycle. With restored regulation of the PAM pathway, advanced breast cancers may become or remain susceptible to endocrine therapy [14].

Natural inhibitors of the PAM pathway are also of interest in developing cancer therapeutics. One such tumor suppressor is phosphatase and tensin homolog (PTEN), which dephosphorylates PIP3 into PIP2, thus effectively reversing PI3K activation [12,15]. The liver kinase B1 (LKB1)-adenosine monophosphate-activated protein kinase (AMPK) pathway is involved in the negative regulation of mTOR signaling, of which upregulation of this pathway would directly target downstream mTOR signaling [13].

### 2.1. mTOR Inhibitors

Everolimus is an mTOR inhibitor that was first approved for use in treating advanced renal cell carcinoma in 2009 and for preventing kidney transplant rejection in 2010 [16]. After evaluating the efficacy of an everolimus-exemestane (an AI) combination in treating hormone receptor (HR)-positive, HER2-negative metastatic breast cancer in postmenopausal women, the US Food and Drug Administration (FDA) approved this drug regimen in 2012 for those who meet the indications [17,18]. Everolimus binds to the FK506-binding protein 12 receptor, and this complex interacts with mTOR to inhibit further “cell cycle progression, cell growth, and proliferation” [17]. Other clinical studies are evaluating the efficacy of everolimus-tamoxifen (a SERM) co-treatment in postmenopausal women with locally advanced/metastatic, HR-positive, HER2-negative, AI-resistant breast cancer. These studies have shown potential clinical benefits in patients with secondary endocrine resistance [14].

Temsirolimus, another mTOR inhibitor similar to everolimus, was approved as an advanced renal cell carcinoma therapeutic by the FDA in 2007 [19]. Temsirolimus also binds to FK506-binding protein, and the resulting complex then binds to mTOR to inhibit its action on cell cycle G1 phase progression, cell growth, and proliferation. Specifically, early clinical studies are evaluating the efficacy of single-agent temsirolimus in ER-positive, HER2-positive, or PTEN-deficient advanced or metastatic breast cancers [20,21]. A phase 2 clinical study of intermittent temsirolimus in combination therapy with daily letrozole, an AI, showed an increased median progression-free survival (PFS) rate compared to letrozole monotherapy in patients with locally advanced/metastatic breast cancer. A following phase 3 clinical study evaluated a similar treatment regimen in patients with HR-positive, locally advanced/metastatic breast cancer who have not been previously exposed to aromatase inhibitors. This study did not show significant clinical benefit, but a subgroup analysis showed increased PFS in patients under the age of 65, compared to patients over 65 years old, thus pointing to a potential for the use of temsirolimus-letrozole combination therapy in younger, postmenopausal breast cancer patients [14].

Sirolimus was also approved by the FDA for usage in preventing organ rejection in kidney transplant patients [16]. A phase 2 clinical trial is studying the efficacy and safety of a sirolimus–tamoxifen dual-drug regimen in HR-positive, HER2-negative metastatic breast cancer. Results so far have shown increased progression-free survival in patients who received the drug combination compared to patients who received single-agent tamoxifen [14].

### 2.2. PI3K Inhibitors

Alpelisib (BYL719), a α-specific PI3K inhibitor, was approved for HR-positive, HER2-negative advanced or metastatic breast cancer treatment in postmenopausal women by the FDA in May 2019. It is typically administered along with fulvestrant, a SERD, to patients with the activating phosphatidylinositol-4,5-bisphosphate 3-kinase catalytic subunit alpha (*PIK3CA*) gene mutations [22]. The *PIK3CA* mutations induce hyperactivation of the PI3K p110 subunit alpha isoform, thus leading to increased cell growth and proliferation. In the SOLAR-1 and BYLieve clinical trials, the synergistic effect of antitumor activity observed in alpelisib–fulvestrant combination therapy resulted in greater PFS, in contrast to usage of either alpelisib or fulvestrant as monotherapies [23,24]. Because approximately 40% of patients with HR-positive, HER2-negative breast cancer also have the *PIK3CA* mutation, alpelisib–fulvestrant is an effective therapeutic combination for targeted therapy, especially for cancers with endocrine resistance [22].

Taselisib (GDC-0032) is a selective class I PI3K inhibitor which spares the p110 subunit beta isoform [25]. It not only blocks PI3K downstream signaling but also induces a decrease in mutated p110α subunit levels. Due to its p110β subunit-sparing characteristic, it is considered to have lower toxicity and greater efficacy than pan-class I PI3K inhibitors [25]. Ongoing clinical trials are studying the efficacy and safety of taselisib in treating advanced breast cancer. SANDPIPER is a phase 3 randomized study which is assessing the clinical utility of taselisib–fulvestrant combination therapy compared to fulvestrant alone in the treatment of ER-positive, HER2-negative, *PIK3CA* mutant advanced or metastatic breast cancer [26]. A phase 1 basket study evaluated the clinical actionability of taselisib in *PIK3CA*-mutant breast cancers, but results showed low clinical actionability of single-agent taselisib therapy [27]. There is also an ongoing phase 1b trial evaluating the safety of taselisib combination therapy with different anti-HER2 drugs in a dose-dependent manner to determine the highest taselisib dose that can be safely administered [25].

Pictilisib (GDC-0941) is an orally available pan-class I PI3K inhibitor under ongoing clinical trials to treat advanced breast cancer [28,29]. Pictilisib binds to the adenosine triphosphate (ATP)-binding pocket, thus non-specifically inhibiting all four isoforms of PI3K: the alpha, beta, delta, and gamma subunits. It was also shown to be effective against *PIK3CA*-mutated, HER2-positive and -negative cancers. Preclinical studies showed increased “antitumor activity of taxanes” and increased apoptosis with pictilisib therapy [29]. In HER2-positive cancers, synergistic cell proliferation inhibition was observed with pictilisib administration together with trastuzumab, a monoclonal antibody used to treat HER2-positive breast cancers. Pictilisib may also have an antiangiogenic ability due to observed growth inhibition when administered to activated human endothelial cells [29]. However, safety has been a notable concern due to its non-isoform-specific actions on PI3K, which can lead to unintended toxicities [28].

Buparlisib (BKM120) is another orally available pan-class I PI3K inhibitor under early phase clinical studies [30]. As with other PI3K inhibitors, clinical trials are evaluating the efficacy and safety of buparlisib in the treatment of endocrine-resistant metastatic breast cancers. A phase 2 study evaluated the efficacy of single-agent therapy with buparlisib in metastatic TNBC and observed no significant prolonged survival [30]. Due to it being a pan-class PI3K inhibitor, dose-limited toxicities include altered mood, rash, and hyperglycemia, thus “highlighting the pharmacological limitations of pan-PI3K inhibition” [28,30].

### 2.3. PTEN Upregulation

Particularly in more aggressive cancers, PTEN tumor suppression is diminished or completely absent, thus contributing to uncontrolled cell growth and proliferation. Because PTEN is a natural inhibitor of the PAM pathway, activation of *PTEN* tumor suppressor expression with the CRISPR/dCas9 system was studied in TNBC to assess a possible therapeutic direction [31]. A study by Moses et al. showed significant inhibition of the PAM pathway downstream signaling through CRISPR/dCas9 induced *PTEN* expression in TNBC cell line SUM159. The activation of *PTEN* expression resulted in decreased phosphorylated Akt and phosphorylated mTOR levels, thus indicating an inhibitory effect on downstream oncogenic signaling [31].

In addition to utilizing gene editing, studies are also being done on natural compounds that have shown potential to be antitumor agents. Breast cancer cell lines MCF-7 and ZR75-1 were treated with Bergapten, a psolaren derivative, to evaluate its anti-survival effects. Results demonstrated increased *PTEN* expression, as well as the induction of autophagy. The triggered autophagy phenotype may increase susceptibility to cell death, thus indicating a possible role for Bergapten administration in inducing breast cancer cell death [32]. In a separate study by Wu et al. on human colon cancer cells, *PTEN* upregulation by oridonin, a Chinese herbal extract, demonstrated the inhibition of cell proliferation and the induction of apoptosis. These colon cancer cells were found to have increased protein levels of PTEN after treatment with oridonin. While this study was performed on colon cancer cells, the results are indicative of antitumor potential from increasing *PTEN* expression in breast cancer cells as well [33].

These findings suggest a possible promising direction for *PTEN* upregulation in future cancer therapeutics. Especially with the precision of the CRISPR/Cas9 system, off-target toxicities will be significantly reduced. In addition, with the increasing prevalence of combined therapy, the use of the CRISPR/Cas9 system along with current breast cancer treatments, such as chemotherapy, endocrine therapy, and/or radiation therapy, could lead to greater efficacy, as well as decreased drug resistance.

### 2.4. LKB1-AMPK Activation

Liver kinase B1 is a serine/threonine kinase that phosphorylates and activates tumor suppressor AMP-activated protein kinase. Activated AMPK then negatively regulates the mTOR pathway, thus arresting further cell growth and metabolism. In addition, activated AMPK inhibits transforming growth factor beta (TGF-β) production and signaling [34]. TGF-β plays a dual role in tumorigenesis where it exerts suppressive effects in the early stages, but then “promotes tumor progression and metastasis in late stages” [35]. One important mechanism by which TGF-β promotes metastasis is through triggering epithelial-to-mesenchymal transition, which causes the cell to gain increased motility, migratory ability, and extracellular matrix degradation ability [34]. Thus, due to the ability of activated AMPK to inhibit TGF-β signaling, this has important implications for AMPK activation as a potential therapeutic target for cancer treatment.

Honokiol is one such agent that activates AMPK through LKB1 phosphorylation. Honokiol is a natural small-molecule polyphenol isolated from the flowering plant species *Magnolia* spp. [36,37]. Cell culture studies using honokiol-treated human breast cancer cell lines MCF7 and MDA-MB-231 showed increased AMPK activation through the LKB1 pathway. This high AMPK activity decreased the migratory and invasive properties of the breast cancer cells, thus demonstrating the ability of the LKB1-AMPK pathway to inhibit breast cancer tumorigenesis [36]. In addition, combination therapy of honokiol with rapamycin showed a synergistic effect on apoptosis induction in breast cancer cells. Thus, honokiol’s ability to target the mTOR pathway further contributes to its potential as a breast cancer therapeutic [37].

## 3. HER2-Positive Targeted Therapies

### 3.1. Tyrosine Kinase Inhibitors

**Figure 2:**
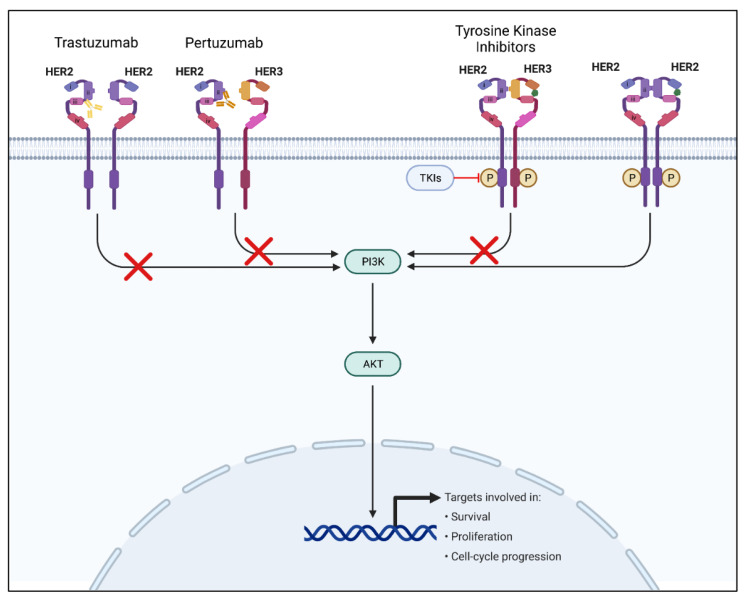
The HER2 signaling pathway and mechanisms of HER2 signaling inhibition. The left-most receptor signaling pathway depicts the mechanism and binding of trastuzumab monoclonal antibody. The receptor pathway second to the left depicts the mechanism and binding of the pertuzumab monoclonal antibody. The receptor pathway second to the right depicts the mechanism of TKIs. Finally, the right-most receptor pathway depicts a normal uninhibited HER2 signaling pathway upon ligand binding. HER2: human epidermal growth factor receptor 2; HER3: human epidermal growth factor receptor 3; TKIs: tyrosine kinase inhibitors; PI3K: Phosphoinositide 3-kinase; Akt: protein kinase B.

HER2-positive breast cancer is a major subtype, making up 20–25% of all breast cancer cases. The prevalence of HER2-positive breast cancer cases makes the HER2 receptor pathway a major point of focus for new and emerging targeted breast cancer therapies [38]. The human epidermal growth factor receptor 2 is a transmembrane protein receptor belonging to the human epidermal growth factor receptor family of tyrosine kinase receptors (EGFR/ERB) [39]. HER2 overexpression is critical in cellular transformation and carcinogenesis in breast cancer and other cancers, including gastric and ovarian cancers [40]. The two main targeted therapies for the HER2 pathway that have been proven effective include monoclonal antibodies and tyrosine kinase inhibitors (TKIs) [41]. Monoclonal antibodies such as trastuzumab target mainly HER2 receptor-binding regions, blocking downstream signaling, whereas tyrosine kinase inhibitors are small molecules that bind and block ATP-binding regions of the HER2 receptor to prevent phosphorylation, and therefore block downstream signaling (Figure 2) [40,41]. Recently, there has been a shift towards developing and using TKIs due to several advantages over monoclonal antibody treatments, including oral administration, decreased cardiotoxicity, and the ability to address multiple targets [41].

Lapatinib was granted the first FDA approval for a TKI in 2007 for cotreatment with capecitabine in HER2-positive/ER-negative/PR-negative breast cancer patients previously treated with standard therapies such as anthracyclines, taxane, and trastuzumab [42]. Lapatinib is a dual inhibitor of HER2 and human epidermal growth factor receptor 1 (HER1) receptors that competitively and reversibly bind their intracellular ATP-binding domains to slow tumor growth. Lapatinib’s effect on the HER1 receptor is negligible in its use for HER2 overexpressing hormone receptor-negative breast cancer patients. In 2010, lapatinib was approved as a first-line treatment with letrozole for postmenopausal hormone and HER2 receptor co-expressing metastatic breast cancer. In 2013, lapatinib’s approval was further extended to include use without chemotherapy in combination with trastuzumab, and following chemotherapy treatment [41,43]. Lapatinib was found to have a synergistic effect when combined with trastuzumab, increasing its apoptotic abilities [44]. The most severe adverse effects of lapatinib were found to be grade 3–4 diarrhea and possible hepatic and cardiac toxicity [45]. Both acquired and inherent resistance to lapatinib were discovered mainly through mutations in the HER2 tyrosine kinase domain, the activation of compensatory pathways, and overexpression or amplification of the gene-encoding trafficking protein particle complex 9 [41].

Neratinib was more recently approved by the FDA in 2017 for the adjuvant treatment of early-stage HER2 overexpressed breast cancer. While lapatinib is a reversible TKI for HER2 and HER1, neratinib is an irreversible TKI for HER1, HER2, and human epidermal growth factor receptor 4 (HER4). Neratinib’s mechanism of action is also slightly different. Instead of competitive inhibition, it induces the covalent linkage of cysteine residues (Cys-773 and Cys-805) to inhibit phosphorylation in the ATP-binding domain of the HER1, HER2, and HER4 receptors [41,46]. Similar to lapatinib, neratinib’s most serious adverse effects were found to be grade 1–3 diarrhea and possible hepatotoxicity [41,47]. Neratinib resistance is still not widely understood; however, connections have been made to the enhanced metabolic activity of cytochrome P450 3A4 [41].

Pyrotinib, an irreversible TKI of HER1, HER2, and HER4, was conditionally approved in 2018 in China for treatment in combination with capecitabine of advanced metastatic HER2-positive breast cancer previously treated with standard anthracycline or taxane chemotherapy [41,48]. Pyrotinib covalently binds to the intracellular receptor ATP-binding domains to inhibit phosphorylation and activation of the downstream pathways. Clinical studies to determine the safety and efficacy of pyrotinib are still ongoing, as well as studies for resistance mechanisms [41].

Most recently, tucatinib received approval by the FDA in 2020 to treat HER2-positive metastatic breast cancer. Compared to the other TKIs, tucatinib is highly selective, proven to be 1000-fold more specific to HER2 than EGFR [38,49]. Tucatinib is also found to have higher central nervous system (CNS) penetration than either lapatinib or neratinib, putting it at the forefront of possible HER2-positive metastatic breast cancer with CNS metastasis treatment [49].

TKIs continue to be a heavily studied category of targeted HER2-positive breast cancer therapy. The potential of TKIs to cross the blood–brain barrier opens the door to the treatment of HER2-positive breast cancer with CNS metastasis, and the increasing implication of TKIs in the treatment and combination treatment of metastatic breast cancer make for a promising outlook for TKI research in the future.

### 3.2. Monoclonal Antibodies

As mentioned in the previous section, monoclonal antibodies are an effective treatment option for HER2-positive breast cancer. Monoclonal antibody treatments have been around for over twenty years, with trastuzumab gaining FDA approval in 1998. Trastuzumab is a first-line treatment option when used alongside chemotherapy for metastatic HER2-positive breast cancer [50].

Trastuzumab binds to the HER2 receptor, blocking downstream cell proliferative signaling through several mechanisms. These mechanisms include the inhibition of heterodimerization (HER2/HER3), homodimerization of the HER2 receptor, and cleavage of the extracellular domain of the HER2 receptor. Dimerization and cleavage are both activating mechanisms of the HER2 signaling cascade. Trastuzumab also helps target HER2-positive cells for destruction by activating the immune system’s antibody-dependent cell-mediated cytotoxicity (ADCC) [51]. Activation of the immune system to attack cancer cells is not unique to HER2-targeted monoclonal antibodies, but is involved in other immunotherapies for cancer we will discuss in a later section [52].

Recent additions to monoclonal antibody regimens for HER2-positive breast cancer have been approved to address resistance mechanisms to trastuzumab and/or increase the efficacy of trastuzumab [51].

Pertuzumab, a monoclonal antibody that binds to the opposite side of the HER2 receptor as trastuzumab, was FDA-approved in late 2017 for use with trastuzumab. Combination therapy of pertuzumab and trastuzumab formed a more complete blockade of the HER2 receptor and were found to have a synergistic effect. Pertuzumab and trastuzumab decreased cell survival by 60% when used together at a dose at which neither individual drug would have any impact. The development of pertuzumab helped address the heterodimerization mechanism of resistance to trastuzumab [51].

Margetuximab, FDA-approved in late 2020 for use with chemotherapy, was introduced to increase immune activation against HER2 positive cells. Margetuximab is specific to the same region of the HER2 receptor as trastuzumab, invoking the same signaling blockade; however, the antibody itself is Fc-engineered to increase affinity for the activating Fcγ receptor and decrease affinity for the FcγR inhibitory receptor. This engineering is proposed to increase both innate and adaptive immune activation against the targeted cell, thereby reducing cell survival [53].

Research into improving the efficacy of monoclonal antibody treatment for HER2-positive breast cancer is ongoing. The domain of the HER2 single-chain variable fragment is of particular interest, as it can be altered to contain dual-specificity including an additional target protein, potentially increasing the antitumor capabilities of monoclonal antibodies [54].

### 3.3. Antibody-Drug Conjugates

Antibody-drug conjugates (ADCs) combine traditional chemotherapy agents with the use of antibodies. Trastuzumab binds to the HER2 receptor, blocking signaling and inducing ADCC to decrease cell survival and proliferation. The currently available antibody-drug conjugates for HER2-positive breast cancer involve trastuzumab conjugated to a chemotherapy drug, synergistically combining the outcomes of both systems [51,55].

Currently, there are two FDA-approved antibody-drug conjugates to treat HER2-positive breast cancer. Trastuzumab emtansine (T-DM1) was the first approved ADC for HER2-positive breast cancer. T-DM1 is composed of a trastuzumab backbone linked via a thioether linker to mertansine, a microtubule inhibitor. Trastuzumab emtansine is a second-line treatment for the treatment of advanced metastatic HER2-positive breast cancer, and was more recently approved for high-risk patients with early-stage residual disease post-neoadjuvant treatment. The ADC was found to have higher efficacy than standard treatment in laboriously pre-treated patients and appeared active in HER2-positive patients with HER2 mutations and variable HER2 expression [55,56].

The second ADC to be approved for the treatment of HER2-positive breast cancer is Trastuzumab deruxtecan (T-DXd). Like T-DM1, T-DXd contains a trastuzumab backbone linked to a chemotherapy drug; however, T-DXd involves a cleavable linker and exatecan with a higher drug to antibody ratio. Exatecan is a topoisomerase inhibitor rather than a microtubule inhibitor like mertansine. Furthermore, the addition of an enzymatically cleavable peptide linker in T-DXd conceivably allows the ADC to be more active even in low HER2-expressing cells, a characteristic not found in T-DM1. Trastuzumab deruxtecan was FDA-approved for the treatment of patients with HER2-positive breast cancer who have been treated with at least two prior HER2-targeting therapies. Both ADCs are accompanied by low-grade adverse effects, including gastrointestinal toxicity and nausea [55,56].

Many HER2-specific ADCs are undergoing clinical trials to become the next-generation of ADC technology for HER2-positive breast cancer treatment. The new ADCs involve novel linkage technologies, as well as a diversity of different payloads. Trastuzumab duocarmazine is notable for its incorporation of its duocarmycin payload in its pro-drug seco-suocarmycin form. Others, such as XMT-1522, are notable for using antibodies with unique epitopes [56].

## 4. HER2-Negative Targeted Therapies

### 4.1. PARP Inhibitors

**Figure 3:**
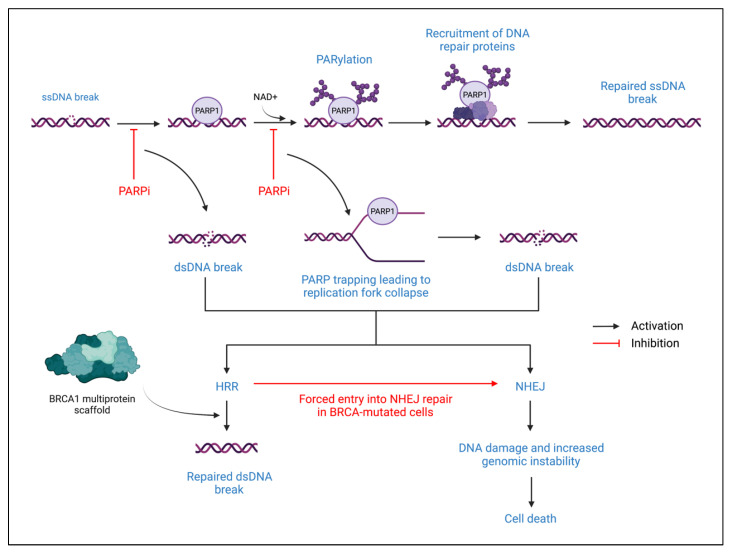
PARP proteins in DNA repair and PARP inhibitor mechanism of action. The top of the figure describes the normal DNA repair mechanism of a single-stranded break. The lower portion of the figure describes how PARP inhibitors alter normal DNA repair. ssDNA: single-stranded DNA; PARP1: poly-ADP-ribose polymerase 1; PARPi: PARP inhibitor; NAD+: nicotinamide adenine dinucleotide; PARylation: poly-ADP-ribosylation; dsDNA: double-stranded DNA; HRR: homologous recombination repair; NHEJ: non-homologous end joining; BRCA: breast cancer-associated protein.

Poly-ADP-Ribose Polymerase (PARP) proteins are involved in deoxyribonucleic acid (DNA) repair processes, primarily through the reversible post-translational modification of nuclear proteins. In addition, PARP proteins also play a role in maintaining genomic stability, thus contributing to cell survival [57]. The two primary PARP proteins are PARP1 and PARP2 which are involved in base excision repair when DNA damage is detected [58]. When DNA damage is sensed, PARP1 will bind to the site of damage and nicotinamide adenine dinucleotide (NAD+) will then bind to the active site on PARP1 (Figure 3). PARP1 induces Poly-ADP-Ribosylation (PARylation) of target nuclear proteins by transferring ADP-ribose moieties from NAD+. This PARylation results in the recruitment of single-strand DNA repair proteins. The release of PARP1 from the site of DNA damage is induced by auto-PARylation, which is followed by a return to a catalytically inactive state [57,58].

In addition to base excision repair, PARP also has a role in homologous recombination repair (HRR). HRR occurs by using a sister chromatid as a template to repair double-strand breaks [58]. When a double-strand break occurs, ataxia telangiectasia-mutated (ATM) kinase and ataxia telangiectasia and Rad3-related kinase recognize the double-strand break and induce signal transduction through phosphorylated CHK2 and Breast Cancer-Associated-1 (BRCA1) proteins. The BRCA1 proteins form a scaffold that organizes DNA repair proteins at the break site, particularly recombinase RAD51, thus facilitating HRR. However, in *BRCA*-mutated cells, HRR is lost due to the inability to form the BRCA1 multiprotein scaffold at the DNA repair site. If a double-strand break occurs, the cell must undergo non-homologous end-joining (NHEJ) DNA repair, typically resulting in cell death due to its error-prone, template-independent mechanism [58]. When a PARP inhibitor is introduced, PARP-mediated DNA repair of single-stranded breaks is inhibited, which stalls the replication fork during DNA replication. This leads to the creation of double-strand breaks, which must be repaired through HRR. Therefore, PARP inhibitors are particularly effective in treating *BRCA*-mutated cancers due to increased cell death susceptibility from forced entry into NHEJ repair after failure to perform the HRR repair of PARP inhibitor-induced double-stranded DNA breakage [58,59,60]. This mechanism of action is formally recognized as synthetic lethality.

Olaparib (AZD-2281) was FDA-approved for monotherapy treatment of HER2-negative metastatic, deleterious germline *BRCA* (*gBRCA*)-mutated breast cancer in 2018 [58,60]. Olaparib specifically targets the catalytic sites of PARP1, PARP2, and PARP3, thus inhibiting PARP activity [59,61]

Talazoparib (BMN-673) is another monotherapy for metastatic/locally advanced HER2-negative, deleterious *gBRCA*-mutated breast cancer that was FDA-approved in 2018 [58]. Talazoparib targets PARP1 and PARP2, and exhibits powerful PARP trapping [59]. Talazoparib competitively binds to the NAD+ binding domain, thus effectively trapping PARP on the DNA at the site of DNA damage. This creates a lesion that stalls replication forks and eventually causes double-stranded breaks to form. The resulting genomic instability caused by talazoparib in *BRCA*-mutated breast cancers is likely how it may cause tumor cell death [61].

Veliparib (ABT-888) is still under ongoing clinical trials and is being evaluated for efficacy in combined treatment with platinum-based chemotherapy for HER2-negative metastatic/locally advanced, *gBRCA*-mutated breast cancer [58]. It targets PARP1 and PARP2 and exhibits weak PARP-trapping capability [59].

Niraparib (MK-4827) targets PARP1 and PARP2. It is currently being evaluated in phase 1 clinical studies for efficacy as neoadjuvant chemotherapy to reduce tumor volume in HER2-negative, *gBRCA*-mutated breast cancers [59].

Rucaparib (AG-014699) is one of the only PARP inhibitors that can target PARP3, in addition to PARP1 and PARP2. It is currently under a phase 2 clinical study evaluating its efficacy as a monotherapeutic agent in patients with *BRCA*-mutated metastatic breast cancer. It is also being studied in a phase 1b/2 clinical study evaluating its safety and efficacy when used in combination with another anticancer agent in patients with triple-negative metastatic breast cancer or *BRCA*-mutated breast cancer [59].

Pamiparib (BGB-290) targets both PARP1 and PARP2. It is being evaluated in a phase 2 study for efficacy and safety as monotherapy in patients with metastatic/locally advanced triple-negative, *BRCA*-mutated breast cancer or just HER2-negative *BRCA*-mutated breast cancer [62].

### 4.2. CDK4/6 Inhibitors

**Figure 4:**
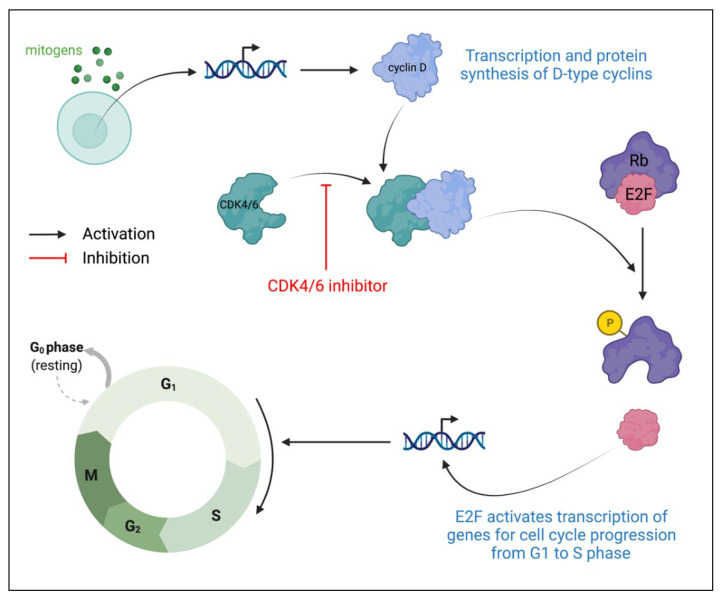
Role of CDK4 and CDK6 in cell growth progression and CDK4/6 inhibitor mechanism of action. CDK: cyclin-dependent kinase; Rb: retinoblastoma protein.

Cyclin-dependent kinases (CDKs) are protein kinases that play prominent roles in cell cycle regulation. CDK4 and CDK6 are G1 kinases that regulate cell cycle exit from the G1 phase and enter the S phase [63,64]. Under the presence of the appropriate growth factors and mitogens, levels of D-type cyclins increase, thus resulting in greater CDK4/6 association with D-type cyclins to create CDK4/6-cyclin D heterodimeric complexes (Figure 4) [65]. These complexes then phosphorylate proteins in the retinoblastoma (Rb) family, causing the release of E2F transcription factors from inhibitory Rb proteins. Released E2F then activates the transcription of genes required for cell cycle progression from G1 to S phase. In HR-positive breast cancers, there is cyclin D overexpression as well as the rare loss of the Rb protein. Thus, due to the ability to target cyclin D, combined with the likely maintenance of the Rb inhibition of E2F, cell cycle progression from G1 to S phase is an ideal therapeutic target in HR-positive breast cancers [66,67,68]. CDK4/6 inhibitors specifically target CDK4 and CDK6, preventing the formation of CDK4/6-cyclin D complexes. Without complex formation, Rb protein will not be phosphorylated to release the E2F transcription factor, causing cell cycle arrest at the G1 phase.

The first-generation CDK4/6 inhibitors demonstrated pan-CDK inhibition, thus limiting their clinical application primarily due to unfavorable, serious adverse effects. One such inhibitor is flavopiridol, which also can only be administered through the intravenous route, thereby complicating its ease of administration, especially in patients with inadequate compliance. It also has demonstrated weak efficacy as a monotherapy, and moderate efficacy when co-treated with other chemotherapy drugs [65]. However, a subsequent generation of CDK inhibitors showed greater selectivity, specifically for CDK4 and CDK6. These CDK inhibitors are palbociclib, ribociclib, and abemaciclib, and can be administered orally, thus bypassing the complexity of intravenous administration [65].

Palbociclib co-treatment with letrozole, an aromatase inhibitor used for hormone-based chemotherapy, was FDA-approved in February 2015. Palbociclib combination therapy with fulvestrant, an ER antagonist, was also FDA-approved in February 2016 for HR-positive, HER2-negative, postmenopausal advanced/metastatic breast cancers [63,69]. Palbociclib combination therapy has shown increased progression-free survival compared to endocrine monotherapy, but it has also demonstrated uncomplicated neutropenia as an adverse effect [69,70,71].

Ribociclib administration with letrozole was also FDA-approved in March 2017 for advanced HR-positive, HER2-negative, postmenopausal breast cancers [63,69]. In addition, ribociclib is being evaluated for efficacy as first and second-line treatment with fulvestrant co-administration in MONALEESA-3, a phase 3 clinical trial [66]. Ribociclib first-line treatment with an aromatase inhibitor may also be indicated in premenopausal women with advanced or metastatic HR-positive, HER2-negative breast cancer [72]. Like palbociclib, ribociclib administration also showed increased progression-free survival and overall survival rate, along with the primary adverse effect of neutropenia, which is fortunately reversible, manageable, and tolerable [71].

Abemaciclib is a selective CDK4/6 inhibitor that inhibits CDK4/cyclin D1 and CDK6/cyclin D1 complexes. Abemaciclib specifically acts as a competitive inhibitor at the ATP-binding domain of CDK4 and CDK6 [65]. It also demonstrates higher selectivity for the CDK4/cyclin D1 complex than palbociclib and ribociclib. Abemaciclib combination therapy with aromatase inhibitors was FDA-approved for HR-positive, HER2-negative, advanced breast cancers in February 2018 [63,69]. In addition, the MONARCH-1 clinical study of abemaciclib monotherapy showed that the single-agent administration of abemaciclib had sufficient efficacy and limited neutropenia toxicity, thus increasing its therapeutic potential among the other specific CDK4/6 inhibitors [63,70].

### 4.3. Antibody-Drug Conjugates

As mentioned in the ADC section above for HER2-positive breast cancer treatments, antibody-drug conjugates consist of a monoclonal antibody linked to a potent chemotherapy agent [55]. Unlike the ADCs used for HER2-positive BC treatment, which involve anti-HER2 antibodies, sacituzumab govitecan (IMMU-132) is composed of an anti-human trophoblast cell-surface antigen 2 (Trop-2) monoclonal antibody, allowing it to target triple-negative breast cancer cells. IMMU-132 is an anti-Trop-2 antibody conjugated to SN-38, a topoisomerase I inhibitor, via a cleavable CL2A linker. Trop-2 is present in breast cancer cells. Therefore, the anti-trop-2 antibody allows IMMU-132 to specifically deliver SN-38 to the breast cancer cells and the surrounding tumor via the cleavable linker [73]. Sacituzumab govitecan was FDA-approved in 2020 for the treatment of metastatic triple-negative breast cancer with a history of two prior metastatic treatments [74].

## 5. Immunotherapy

### 5.1. Immune Checkpoint Inhibitors

**Figure 5:**
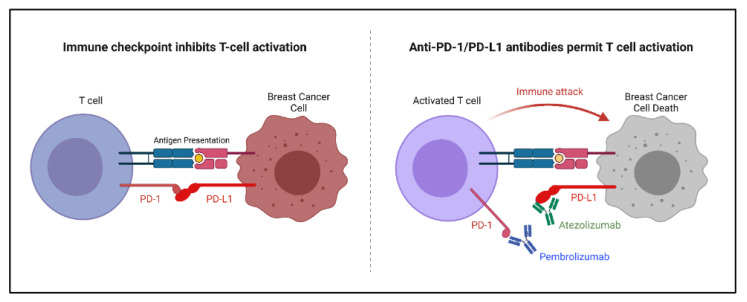
Mechanism of immune checkpoint inhibitors pembrolizumab and atezolizumab on the PD-1/PD-L1 axis. PD-1: programmed cell death protein 1; PD-L1: programmed cell death ligand 1.

The role of the immune system in breast cancer treatment is currently being thoroughly explored. It has been shown that the activation of tumor infiltrating lymphocytes can lead to a better breast cancer prognosis [52]. Immune checkpoint inhibitors were developed to increase the immune response, specifically by activating cytotoxic T lymphocytes against active tumors. Immune checkpoint inhibitors in breast cancer target the programmed cell death protein 1/programmed cell death ligand 1 (PD-1/PD-L1) axis due to its impact specifically on breast cancer (Figure 5). Other immune checkpoints, such as the cytotoxic T-lymphocyte-associated antigen 4 checkpoint, are less significant in breast cancer. The PD-1/PD-L1 interaction inhibits cytotoxic T cell activation as a regulatory mechanism. A blockade of the PD-1/PD-L1 axis allows for an increase in the activation of cytotoxic T lymphocytes which become available to infiltrate and attack the breast cancer tumor [52,75].

Several monoclonal antibodies have been designed to bind and block the PD-1/PD-L1 axis. Two of these antibodies in particular have been found to be effective when combined with chemotherapy for advanced triple-negative breast cancer. Specifically, atezolizumab (PD-L1-binding) and pembrolizumab (PD-1-binding) had only minimal impact when used alone against heavily pretreated TNBC patients; however, upon the addition of chemotherapy, both antibodies demonstrated a significant increase in efficacy. Both atezolizumab and pembrolizumab have been approved for use in advanced stage TNBC patients [75,76].

Other monoclonal antibodies that target the PD-1/PD-L1 interaction include durvalumab and nivolumab. These antibodies have demonstrated effectiveness against other cancers such as small cell lung cancer; however, they have not been approved for breast cancer. Durvalumab has shown promise in combination with chemotherapy against early-stage TNBC, but has yet to be approved for this use [75].

Immune checkpoint inhibitors are a novel treatment option for breast cancer and continue to be investigated and improved [52,75].

### 5.2. Cancer Vaccines

Another rising area of research in breast cancer treatment is the therapeutic potential of cancer vaccines. Current research and clinical studies aim to evaluate the efficacy of cancer vaccines in cancer treatment and the prevention of cancer recurrence [77,78]. Cancer vaccines aim to mobilize the patient’s own immune system to stimulate cytotoxic T-lymphocytes that target the tumor and stimulate the production of long-term memory cluster of differentiation 8 positive (cytotoxic) T cells to prevent recurrence [52,78,79]. In addition, vaccines do not need to be administered as frequently as traditional cancer therapeutics, and generally have less toxicity when compared to chemotherapy [80]. Among cancer vaccine research, peptide vaccines are the primary focus of interest in breast cancer vaccines. Peptide vaccines aim to introduce specific tumor antigens that are not found in normal tissue, thereby stimulating the immune system to recognize and target these specific antigens in cancer cells [52,80,81]. Many ongoing clinical trials are evaluating the efficacy and safety of cancer vaccines in adjuvant and neoadjuvant settings, especially for HER2-positive and aggressive triple-negative breast cancers [81].

A breast cancer vaccine that is being extensively researched is the E75 peptide vaccine, which is also known as nelipepimut-S. E75 introduces a nine amino acid peptide that is “derived from the extracellular domain of the HER2 protein”, thus targeting HER2-positive breast cancers [82]. E75 is expected to activate the cytotoxic T-lymphocyte response by binding to the human leukocyte antigen-A2 (HLA-A2) serotype of major histocompatibility complex class 1 glycoproteins [79]. A phase 3 clinical trial (PRESENT) studied the efficacy of E75 co-administration with granulocyte-macrophage colony-stimulating factor (GM-CSF) immunoadjuvant in preventing breast cancer recurrence in an adjuvant setting [81]. However, E75 co-treatment with GM-CSF did not demonstrate any therapeutic benefit in preventing cancer recurrence [79,80].

GP2 is another breast cancer vaccine that was evaluated for efficacy in decreasing the rate of recurrence in patients with HER2-positive breast cancer. GP2 is derived from a nine amino acid peptide of the HER2 protein transmembrane domain [79,80]. Similar to E75, GP2 is predicted to bind to HLA-A2 to activate cytotoxic T-lymphocytes, but with lesser affinity than E75. While the vaccine demonstrated clinical safety, it also did not demonstrate any significant therapeutic benefit [79]. However, other peptide vaccines targeting different tumor antigens are being studied, both as monotherapies and in combination therapies, in hope of discovering cancer vaccines that are effective both prophylactically and therapeutically [79,80,81].

In December 2020, the FDA approved the investigation of a prophylactic TNBC vaccine developed by Cleveland Clinic’s Dr. Vincent Tuohy. In collaboration with Anixa Biosciences, phase 1 clinical trials will evaluate the efficacy of this vaccine in postmenopausal patients with high-risk, early-stage TNBC. This vaccine specifically introduces alpha-lactalbumin, a protein which is expressed in the mammary glands only during lactation. Alpha-lactalbumin was found to be abnormally expressed at high levels in breast cancer cells, especially in TNBC. Thus, this vaccine may have significant prophylactic and therapeutic potential in postmenopausal women [83].

In addition to peptide vaccines, there are also ongoing clinical trials studying the efficacies of whole protein vaccines, bacterial/viral vaccines, cell-based vaccines, and gene-based vaccines in breast cancer treatment. Whole protein vaccines may be more advantageous compared to peptide vaccines due to the ability to bind both HLA class I and II epitopes, thereby bypassing specific HLA restrictions. Viral vaccines can be used to infect antigen-presenting cells (APCs) and induce the expression of transgenes specifically found in tumor cells. In addition, using certain oncolytic viruses against tumor cells can add to its therapeutic potential [80]. Cell-based vaccines revolve around introducing autologous tumor-cell based vaccines or allogeneic tumor-cell-based vaccines to induce an immune response against a repertoire of tumor-associated antigens (TAAs). Gene-based vaccines, such as DNA vaccines, are being designed to transfect APCs and induce the expression of TAAs in these transfected APCs. Through this mechanism, APCs can mount a potent and specific immune response against TAAs encoded in the DNA vaccine [79,80].

## 6. Conclusions

Recently, there has been much progress in treatment discovery for all subtypes of breast cancer, spanning a diversity of mechanisms from signaling blockades to immune system mobilization through vaccination. The expansion of targeted and immune therapies for breast cancer has greatly increased treatment options, especially for late-stage advanced breast cancers. With many new breast cancer drug approvals surfacing in just the last few years, it is clear there is still much to look forward to for the future of breast cancer treatment. The targeted therapies we discussed have changed the outlook of breast cancer treatment, and created hope for breast cancer patients who are still struggling to find a cure.

## Figures and Tables

**Table 1:** Emerging and novel breast cancer drugs discussed in this article organized by breast cancer subtype target.

Breast Cancer Subtype	Drug Category	Drug Name	Patient Population	Therapy Given
HR-positive	mTOR Inhibitors	Everolimus	HR+, HER2− Postmenopausal	Everolimus + Exemestane
			HR+, HER2− Postmenopausal AI-resistant	Everolimus + Tamoxifen
		Temsirolimus	ER+, HER2+ PTEN-deficient	Single-agent temsirolimus
			ER+, HER2+	Temsirolimus + Letrozole
		Sirolimus	HR+, HER2−	Sirolimus + Tamoxifen
	PI3K Inhibitors	Alpelisib	HR+, HER2− *PIK3CA* mutant Postmenopausal	Alpelisib + Fulvestrant
		Taselisib	ER+, HER2− *PIK3CA* mutant	Taselisib + Fulvestrant
		Pictilisib	HER2+/− *PIK3CA* mutant	Pictilisib + Trastuzumab
		Buparlisib	ER−, PR−, HER2− (TNBC)	Single-agent buparlisib
HER2-positive	TKIs	Lapatinib	ER−, PR−, HER2+	Lapatinib + Capecitabine
			HR+, HER2+	Lapatinib + Letrozole
			HR+, HER2+	Lapatinib + Trastuzumab
		Neratinib	HER2+ Early-stage	Single-agent neratinib
		Pyrotinib	HER2+	Pyrotinib + Capecitabine
		Tucatinib	HER2+	Single-agent tucatinib
	Monoclonal Antibodies	Trastuzumab	HER2+	Single-agent trastuzumab
		Pertuzumab	HER2+	Pertuzumab + Trastuzumab
		Margetuximab	HER2+	Margetuximab + chemotherapy
	Antibody-drug Conjugates	Trastuzumab Emtansine (T-DM1)	HER2+ Early-stage residual disease Post-neoadjuvant tx	Single-agent T-DM1
		Trastuzumab Deruxtecan (T-DXd)	HER2+ Tx with at least two prior HER2-targeted therapies	Single-agent T-DXd
HER2-negative	PARP Inhibitors	Olaparib	HER2− Deleterious *gBRCA* mutant	Single-agent Olaparib
		Talazoparib	HER2− Deleterious *gBRCA* mutant	Single-agent talazoparib
		Veliparib	HER2− *gBRCA* mutant	Veliparib + platinum-based chemotherapy
		Niraparib	HER2− *gBRCA* mutant	Niraparib as neoadjuvant chemotherapy
		Rucaparib	*gBRCA* mutant	Single-agent rucaparib
			*gBRCA* mutant or TNBC	Rucaparib + anticancer agent
		Pamiparib	TNBC, *gBRCA* muant or HER2−, *gBRCA* mutant	Single-agent pamiparib
	CDK4/CDK6 Inhibitors	Palbociclib	HER2−	Palbociclib + Letrozole
			HR+, HER2− Postmenopausal	Palbociclib + Fulvestrant
		Ribociclib	HR+, HER2− Post/premenopausal	Ribociclib + Letrozole
			HR+, HER2− Postmenopausal	Ribociclib + Fulvestrant
		Abemaciclib	HR+, HER2−	Abemaciclib + AI
			HR+, HER2−	Single-agent abemaciclib
	Antibody-drug Conjugates	Sacituzumab Govitecan (IMMU-132)	TNBC Hx of two prior metastatic tx	Single-agent IMMU-132
TNBC	Immune Checkpoint Inhibitors	Atezolizumab	TNBC	Atezolizumab + chemotherapy
		Pembrolizumab	TNBC	Pembrolizumab + chemotherapy
	Cancer Vaccines	E75	HER2+	E75 + GM-CSF
		GP2	HER2+	Single-agent GP2
Other	LKB1-AMPK Pathway Activator	Honokiol	Endocrine-resistant BC	Honokiol + rapamycin

HR: hormone receptor; ER: estrogen receptor; PR: progesterone receptor; HER2: human epidermal growth factor receptor 2; TNBC: triple-negative breast cancer; mTOR: mammalian target of rapamycin; PI3K: phosphoinositide 3-kinase; TKI: tyrosine kinase inhibitor; PARP: poly-ADP-ribose polymerase; CDK: cyclin-dependent kinase; LKB1: liver kinase B1; AMPK: adenosine monophosphate-activated protein kinase; T-DM1: trastuzumab emtansine; T-DXd: trastuzumab deruxtecan; IMMU-132: sacituzumab govitecan; PTEN: phosphatase and tensin homolog; *PIK3CA*: phosphatidylinositol-4,5-bisphosphate 3-kinase catalytic subunit alpha gene; tx: treatment; hx: history; *gBRCA*: germline breast cancer-associated protein; BC: breast cancer; AI: aromatase inhibitor; GM-CSF: granulocyte-macrophage colony-stimulating factor.

## Data Availability

This study did not report any data.

## Abbreviations

ADC	Antibody-drug conjugate
ADCC	Antibody-dependent cell-mediated cytotoxicity
AI	Aromatase inhibitor
Akt	Protein kinase B
AMPK	Adenosine monophosphate-activated protein kinase
APC	Antigen-presenting cell
ATM	Ataxia telangiectasia-mutated kinase
ATP	Adenosine triphosphate
BC	Breast cancer
BRCA	Breast cancer-associated protein
CDK	Cyclin-dependent kinase
CK	Cytokeratins
CNS	Central nervous system
DNA	Deoxyribonucleic acid
dsDNA	Double-stranded deoxyribonucleic acid
EGFR/ERB	Epidermal growth factor receptor
ER	Estrogen receptor
FDA	Food and drug administration
*gBRCA*	Germline breast cancer associated protein gene
GM-CSF	Granulocyte-macrophage colony-stimulating factor
HER1	Human epidermal growth factor receptor 1
HER2	Human epidermal growth factor receptor 2
HER3	Human epidermal growth factor receptor 3
HER4	Human epidermal growth factor receptor 4
HLA-A2	Human leukocyte antigen-A2
HR	Hormone receptor
HRR	Homologous recombination repair
LKB1	Liver kinase B1
LumA	Luminal-A
LumB	Luminal-B
mTOR	Mammalian target of rapamycin
NAD+	Nicotinamide adenine dinucleotide
NHEJ	Non-homologous end joining
PAM	PI3K/Akt/mTOR pathway
PARP	Poly-ADP-ribose polymerase
PARPi	Poly-ADP-ribose polymerase inhibitor
PARylation	Poly-ADP-ribosylation
*PIK3CA*	Phosphatidylinositol-4,5-bisphosphate 3-kinase catalytic subunit alpha gene
PIP2	Phosphatidylinositol-4,5-biphosphate
PIP3	Phosphatidylinositol-3,4,5-triphosphate
PI3K	Phosphoinositide 3-kinase
PR	Progesterone receptor
PD-1	Programmed cell death protein 1
PD-L1	Programmed cell death ligand 1
PTEN	Phosphatase and tensin homolog
Rb	Retinoblastoma protein
RTK	Receptor tyrosine kinase
SERD	Selective estrogen receptor degrader
SERM	Selective estrogen receptor modulator
TAA	Tumor-associated antigen
T-DM1	Trastuzumab emtansine
T-DXd	Trastuzumab deruxtecan
TGF-β	Transforming growth factor beta
TKI	Tyrosine kinase inhibitor
TNBC	Triple-negative breast cancer
Trop-2	Trophoblast cell-surface antigen 2

## References

[1] Siegel R.L., Miller K.D., Fuchs H.E., Jemal A. (2021). Cancer Statistics, 2021. CA Cancer J. Clin..

[2] Breast Cancer Facts & Figures 2019–2020. https://www.cancer.org/research/cancer-facts-statistics/breast-cancer-facts-figures.html.

[3] Li C., Fan Z., Lin X., Cao M., Song F., Song F. (2021). Parity and risk of developing breast cancer according to tumor subtype: A systematic review and meta-analysis. Cancer Epidemiol..

[4] Carey L.A., Perou C.M., Livasy C.A., Dressler L.G., Cowan D., Conway K., Karaca G., Troester M.A., Tse C.K., Edmiston S. (2006). Race, breast cancer subtypes, and survival in the Carolina Breast Cancer Study. JAMA.

[5] Haque R., Ahmed S.A., Inzhakova G., Shi J., Avila C., Polikoff J., Bernstein L., Enger S.M., Press M.F. (2012). Impact of breast cancer subtypes and treatment on survival: An analysis spanning two decades. Cancer Epidemiol. Biomark. Prev..

[6] Incidence and Relative Survival by Stage at Diagnosis for Common Cancers. https://www.cdc.gov/cancer/uscs/about/data-briefs/no25-incidence-relative-survival-stage-diagnosis.htm.

[7] Elston C.W., Ellis I.O. (1991). Pathological prognostic factors in breast cancer. I. The value of histological grade in breast cancer: Experience from a large study with long-term follow-up. Histopathology.

[8] Harbeck N., Gnant M. (2017). Breast cancer. Lancet.

[9] Koh S.B., Ellisen L.W. (2021). Immune activation and evolution through chemotherapy plus checkpoint blockade in triple-negative breast cancer. Cancer Cell.

[10] Shanmugam G., Rakshit S., Sarkar K. (2022). HDAC inhibitors: Targets for tumor therapy, immune modulation and lung diseases. Transl. Oncol..

[11] Manso L., Salvador F., Villagrasa P., Chic N., Bermejo B., Cejalvo J.M., Izarzugaza Y., Cantos B., Blanch S., Margeli M. (2021). Abstract CT191: A window-of-opportunity study with atezolizumab and the oncolytic virus pelareorep in early breast cancer (AWARE-1). Cancer Res..

[12] Paplomata E., O’Regan R. (2014). The PI3K/AKT/mTOR pathway in breast cancer: Targets, trials and biomarkers. Ther. Adv. Med. Oncol..

[13] Chang F., Lee J.T., Navolanic P.M., Steelman L.S., Shelton J.G., Blalock W.L., Franklin R.A., McCubrey J.A. (2003). Involvement of PI3K/Akt pathway in cell cycle progression, apoptosis, and neoplastic transformation: A target for cancer chemotherapy. Leukemia.

[14] Arena F. (2014). Clinical implications of recent studies using mTOR inhibitors to treat advanced hormone receptor-positive breast cancer. Cancer Manag. Res..

[15] Carbognin L., Miglietta F., Paris I., Dieci M.V. (2019). Prognostic and Predictive Implications of PTEN in Breast Cancer: Unfulfilled Promises but Intriguing Perspectives. Cancers.

[16] Steelman L.S., Martelli A.M., Cocco L., Libra M., Nicoletti F., Abrams S.L., McCubrey J.A. (2016). The therapeutic potential of mTOR inhibitors in breast cancer. Br. J. Clin. Pharmacol..

[17] Royce M.E., Osman D. (2015). Everolimus in the Treatment of Metastatic Breast Cancer. Breast Cancer.

[18] Riccardi F., Colantuoni G., Diana A., Mocerino C., Carteni G., Lauria R., Febbraro A., Nuzzo F., Addeo R., Marano O. (2018). Exemestane and Everolimus combination treatment of hormone receptor positive, HER2 negative metastatic breast cancer: A retrospective study of 9 cancer centers in the Campania Region (Southern Italy) focused on activity, efficacy and safety. Mol. Clin. Oncol..

[19] Kwitkowski V.E., Prowell T.M., Ibrahim A., Farrell A.T., Justice R., Mitchell S.S., Sridhara R., Pazdur R. (2010). FDA approval summary: Temsirolimus as treatment for advanced renal cell carcinoma. Oncologist.

[20] Chan S., Scheulen M.E., Johnston S., Mross K., Cardoso F., Dittrich C., Eiermann W., Hess D., Morant R., Semiglazov V. (2005). Phase II study of temsirolimus (CCI-779), a novel inhibitor of mTOR, in heavily pretreated patients with locally advanced or metastatic breast cancer. J. Clin. Oncol..

[21] Fleming G.F., Ma C.X., Huo D., Sattar H., Tretiakova M., Lin L., Hahn O.M., Olopade F.O., Nanda R., Hoffman P.C. (2012). Phase II trial of temsirolimus in patients with metastatic breast cancer. Breast Cancer Res. Treat..

[22] Andre F., Ciruelos E., Rubovszky G., Campone M., Loibl S., Rugo H.S., Iwata H., Conte P., Mayer I.A., Kaufman B. (2019). Alpelisib for PIK3CA-Mutated, Hormone Receptor-Positive Advanced Breast Cancer. N. Engl. J. Med..

[23] Rugo H.S., Lerebours F., Ciruelos E., Drullinsky P., Ruiz-Borrego M., Neven P., Park Y.H., Prat A., Bachelot T., Juric D. (2021). Alpelisib plus fulvestrant in PIK3CA-mutated, hormone receptor-positive advanced breast cancer after a CDK4/6 inhibitor (BYLieve): One cohort of a phase 2, multicentre, open-label, non-comparative study. Lancet Oncol..

[24] Turner S., Chia S., Kanakamedala H., Hsu W.C., Park J., Chandiwana D., Ridolfi A., Yu C.L., Zarate J.P., Rugo H.S. (2021). Effectiveness of Alpelisib + Fulvestrant Compared with Real-World Standard Treatment Among Patients with HR+, HER2-, PIK3CA-Mutated Breast Cancer. Oncologist.

[25] Janku F. (2017). Phosphoinositide 3-kinase (PI3K) pathway inhibitors in solid tumors: From laboratory to patients. Cancer Treat. Rev..

[26] Dent S., Cortes J., Im Y.H., Dieras V., Harbeck N., Krop I.E., Wilson T.R., Cui N., Schimmoller F., Hsu J.Y. (2021). Phase III randomized study of taselisib or placebo with fulvestrant in estrogen receptor-positive, PIK3CA-mutant, HER2-negative, advanced breast cancer: The SANDPIPER trial. Ann. Oncol..

[27] Jhaveri K., Chang M.T., Juric D., Saura C., Gambardella V., Melnyk A., Patel M.R., Ribrag V., Ma C.X., Aljumaily R. (2021). Phase I Basket Study of Taselisib, an Isoform-Selective PI3K Inhibitor, in Patients with PIK3CA-Mutant Cancers. Clin. Cancer Res..

[28] Zhang M., Jang H., Nussinov R. (2020). PI3K inhibitors: Review and new strategies. Chem. Sci..

[29] Schoffski P., Cresta S., Mayer I.A., Wildiers H., Damian S., Gendreau S., Rooney I., Morrissey K.M., Spoerke J.M., Ng V.W. (2018). A phase Ib study of pictilisib (GDC-0941) in combination with paclitaxel, with and without bevacizumab or trastuzumab, and with letrozole in advanced breast cancer. Breast Cancer Res..

[30] Garrido-Castro A.C., Saura C., Barroso-Sousa R., Guo H., Ciruelos E., Bermejo B., Gavila J., Serra V., Prat A., Pare L. (2020). Phase 2 study of buparlisib (BKM120), a pan-class I PI3K inhibitor, in patients with metastatic triple-negative breast cancer. Breast Cancer Res..

[31] Moses C., Nugent F., Waryah C.B., Garcia-Bloj B., Harvey A.R., Blancafort P. (2019). Activating PTEN Tumor Suppressor Expression with the CRISPR/dCas9 System. Mol. Ther. Nucleic Acids.

[32] De Amicis F., Aquila S., Morelli C., Guido C., Santoro M., Perrotta I., Mauro L., Giordano F., Nigro A., Ando S. (2015). Bergapten drives autophagy through the up-regulation of PTEN expression in breast cancer cells. Mol. Cancer.

[33] Wu Q.X., Yuan S.X., Ren C.M., Yu Y., Sun W.J., He B.C., Wu K. (2016). Oridonin upregulates PTEN through activating p38 MAPK and inhibits proliferation in human colon cancer cells. Oncol. Rep..

[34] Li N., Huang D., Lu N., Luo L. (2015). Role of the LKB1/AMPK pathway in tumor invasion and metastasis of cancer cells (Review). Oncol. Rep..

[35] Li N.S., Zou J.R., Lin H., Ke R., He X.L., Xiao L., Huang D., Luo L., Lv N., Luo Z. (2016). LKB1/AMPK inhibits TGF-beta1 production and the TGF-beta signaling pathway in breast cancer cells. Tumor Biol..

[36] Nagalingam A., Arbiser J.L., Bonner M.Y., Saxena N.K., Sharma D. (2012). Honokiol activates AMP-activated protein kinase in breast cancer cells via an LKB1-dependent pathway and inhibits breast carcinogenesis. Breast Cancer Res..

[37] Arora S., Singh S., Piazza G.A., Contreras C.M., Panyam J., Singh A.P. (2012). Honokiol: A novel natural agent for cancer prevention and therapy. Curr. Mol. Med..

[38] Schlam I., Swain S.M. (2021). HER2-positive breast cancer and tyrosine kinase inhibitors: The time is now. NPJ Breast Cancer.

[39] Mitri Z., Constantine T., O’Regan R. (2012). The HER2 Receptor in Breast Cancer: Pathophysiology, Clinical Use, and New Advances in Therapy. Chemother. Res. Pract..

[40] Yu S., Liu Q., Han X., Qin S., Zhao W., Li A., Wu K. (2017). Development and clinical application of anti-HER2 monoclonal and bispecific antibodies for cancer treatment. Exp. Hematol. Oncol..

[41] Xuhong J.C., Qi X.W., Zhang Y., Jiang J. (2019). Mechanism, safety and efficacy of three tyrosine kinase inhibitors lapatinib, neratinib and pyrotinib in HER2-positive breast cancer. Am. J. Cancer Res..

[42] Ryan Q., Ibrahim A., Cohen M.H., Johnson J., Ko C.W., Sridhara R., Justice R., Pazdur R. (2008). FDA drug approval summary: Lapatinib in combination with capecitabine for previously treated metastatic breast cancer that overexpresses HER-2. Oncologist.

[43] Voigtlaender M., Schneider-Merck T., Trepel M. (2018). Lapatinib. Recent Results Cancer Res..

[44] Incorvati J.A., Shah S., Mu Y., Lu J. (2013). Targeted therapy for HER2 positive breast cancer. J. Hematol. Oncol..

[45] Gomez H.L., Doval D.C., Chavez M.A., Ang P.C., Aziz Z., Nag S., Ng C., Franco S.X., Chow L.W., Arbushites M.C. (2008). Efficacy and safety of lapatinib as first-line therapy for ErbB2-amplified locally advanced or metastatic breast cancer. J. Clin. Oncol..

[46] Oliveira M., Garrigos L., Assaf J.D., Escriva-de-Romani S., Saura C. (2020). Neratinib plus capecitabine for the treatment of advanced HER2-positive breast cancer. Expert Rev. Anticancer Ther..

[47] (2019). Neratinib for breast cancer. Aust. Prescr..

[48] Blair H.A. (2018). Pyrotinib: First Global Approval. Drugs.

[49] Ulrich L., Okines A.F.C. (2021). Treating Advanced Unresectable or Metastatic HER2-Positive Breast Cancer: A Spotlight on Tucatinib. Breast Cancer.

[50] Wuerstlein R., Harbeck N. (2017). Neoadjuvant Therapy for HER2-positive Breast Cancer. Rev. Recent Clin. Trials.

[51] Richard S., Selle F., Lotz J.P., Khalil A., Gligorov J., Soares D.G. (2016). Pertuzumab and trastuzumab: The rationale way to synergy. An. Acad. Bras. Cienc..

[52] Li Y., Miao W., He D., Wang S., Lou J., Jiang Y., Wang S. (2021). Recent Progress on Immunotherapy for Breast Cancer: Tumor Microenvironment, Nanotechnology and More. Front. Bioeng. Biotechnol..

[53] Rugo H.S., Im S.A., Cardoso F., Cortes J., Curigliano G., Musolino A., Pegram M.D., Wright G.S., Saura C., Escriva-de-Romani S. (2021). Efficacy of Margetuximab vs Trastuzumab in Patients with Pretreated ERBB2-Positive Advanced Breast Cancer: A Phase 3 Randomized Clinical Trial. JAMA Oncol..

[54] Costa R.L.B., Czerniecki B.J. (2020). Clinical development of immunotherapies for HER2^+^ breast cancer: A review of HER2-directed monoclonal antibodies and beyond. NPJ Breast Cancer.

[55] Nakada T., Sugihara K., Jikoh T., Abe Y., Agatsuma T. (2019). The Latest Research and Development into the Antibody-Drug Conjugate, [fam-] Trastuzumab Deruxtecan (DS-8201a), for HER2 Cancer Therapy. Chem. Pharm. Bull..

[56] Ferraro E., Drago J.Z., Modi S. (2021). Implementing antibody-drug conjugates (ADCs) in HER2-positive breast cancer: State of the art and future directions. Breast Cancer Res..

[57] Herceg Z., Wang Z.Q. (2001). Functions of poly(ADP-ribose) polymerase (PARP) in DNA repair, genomic integrity and cell death. Mutat. Res..

[58] Cortesi L., Rugo H.S., Jackisch C. (2021). An Overview of PARP Inhibitors for the Treatment of Breast Cancer. Target. Oncol..

[59] Goncalves A., Bertucci A., Bertucci F. (2020). PARP Inhibitors in the Treatment of Early Breast Cancer: The Step Beyond?. Cancers.

[60] Goulooze S.C., Cohen A.F., Rissmann R. (2016). Olaparib. Br. J. Clin. Pharmacol..

[61] Rose M., Burgess J.T., O’Byrne K., Richard D.J., Bolderson E. (2020). PARP Inhibitors: Clinical Relevance, Mechanisms of Action and Tumor Resistance. Front. Cell Dev. Biol..

[62] Sun T., Shi Y., Cui J., Yin Y., Ouyang Q., Liu Q., Zhang Q., Chen Y., Zhimin S., Wang S. (2021). A phase 2 study of pamiparib in the treatment of patients with locally advanced or metastatic HER2-negative breast cancer with germline *BRCA* mutation. J. Clin. Oncol..

[63] Tigan A.S., Bellutti F., Kollmann K., Tebb G., Sexl V. (2016). CDK6-a review of the past and a glimpse into the future: From cell-cycle control to transcriptional regulation. Oncogene.

[64] Du Q., Guo X., Wang M., Li Y., Sun X., Li Q. (2020). The application and prospect of CDK4/6 inhibitors in malignant solid tumors. J. Hematol. Oncol..

[65] Corona S.P., Generali D. (2018). Abemaciclib: A CDK4/6 inhibitor for the treatment of HR+/HER2- advanced breast cancer. Drug Des. Dev. Ther..

[66] Shah M., Nunes M.R., Stearns V. (2018). CDK4/6 Inhibitors: Game Changers in the Management of Hormone Receptor-Positive Advanced Breast Cancer?. Oncology.

[67] Abraham J., Coleman R., Elias A., Holmes F.A., Kalinsky K., Kittaneh M., Lower E., Mahtani R., Terry Mamounas E., Pegram M. (2018). Use of cyclin-dependent kinase (CDK) 4/6 inhibitors for hormone receptor-positive, human epidermal growth factor receptor 2-negative, metastatic breast cancer: A roundtable discussion by The Breast Cancer Therapy Expert Group (BCTEG). Breast Cancer Res. Treat..

[68] O’Leary B., Finn R.S., Turner N.C. (2016). Treating cancer with selective CDK4/6 inhibitors. Nat. Rev. Clin. Oncol..

[69] Bayraktar S., Batoo S., Al-Hattab E., Basu S., Okuno S., Gluck S. (2020). Future perspectives and challenges with CDK4/6 inhibitors in hormone receptor-positive metastatic breast cancer. Future Oncol..

[70] Shah A.N., Cristofanilli M. (2017). The Growing Role of CDK4/6 Inhibitors in Treating Hormone Receptor-Positive Advanced Breast Cancer. Curr. Treat. Options Oncol..

[71] Li J., Fu F., Yu L., Huang M., Lin Y., Mei Q., Lv J., Wang C. (2020). Cyclin-dependent kinase 4 and 6 inhibitors in hormone receptor-positive, human epidermal growth factor receptor-2 negative advanced breast cancer: A meta-analysis of randomized clinical trials. Breast Cancer Res. Treat..

[72] Cersosimo R.J. (2019). Cyclin-dependent kinase 4/6 inhibitors for the management of advanced or metastatic breast cancer in women. Am. J. Health Syst. Pharm..

[73] Bardia A., Mayer I.A., Vahdat L.T., Tolaney S.M., Isakoff S.J., Diamond J.R., O’Shaughnessy J., Moroose R.L., Santin A.D., Abramson V.G. (2019). Sacituzumab Govitecan-hziy in Refractory Metastatic Triple-Negative Breast Cancer. N. Engl. J. Med..

[74] Syed Y.Y. (2020). Sacituzumab Govitecan: First Approval. Drugs.

[75] Schmidt M., Heimes A.S. (2021). Immunomodulating Therapies in Breast Cancer-From Prognosis to Clinical Practice. Cancers.

[76] Lee H.T., Lee S.H., Heo Y.S. (2019). Molecular Interactions of Antibody Drugs Targeting PD-1, PD-L1, and CTLA-4 in Immuno-Oncology. Molecules.

[77] Behravan J., Razazan A., Behravan G. (2019). Towards Breast Cancer Vaccines, Progress and Challenges. Curr. Drug Discov. Technol..

[78] Solinas C., Aiello M., Migliori E., Willard-Gallo K., Emens L.A. (2020). Breast cancer vaccines: Heeding the lessons of the past to guide a path forward. Cancer Treat. Rev..

[79] Pallerla S., Abdul A., Comeau J., Jois S. (2021). Cancer Vaccines, Treatment of the Future: With Emphasis on HER2-Positive Breast Cancer. Int. J. Mol. Sci..

[80] Corti C., Giachetti P., Eggermont A.M.M., Delaloge S., Curigliano G. (2022). Therapeutic vaccines for breast cancer: Has the time finally come?. Eur. J. Cancer.

[81] Criscitiello C., Viale G., Curigliano G. (2019). Peptide vaccines in early breast cancer. Breast.

[82] Clifton G.T., Peoples G.E., Mittendorf E.A. (2016). The development and use of the E75 (HER2 369-377) peptide vaccine. Future Oncol..

[83] Anixa Biosciences and Cleveland Clinic File IND Application for Breast Cancer Vaccine. https://www.biospace.com/article/releases/anixa-biosciences-and-cleveland-clinic-file-ind-application-for-breast-cancer-vaccine/.

